# A Southwestern United States Pilot Investigation of Triatomine–Mite Prevalence

**DOI:** 10.3390/insects12090811

**Published:** 2021-09-10

**Authors:** Kyndall C. Dye-Braumuller, Hanna Waltz, Mary K. Lynn, Stephen A. Klotz, Justin O. Schmidt, Alvaro Romero, Marvin Stanley Rodriguez Aquino, Jose Ricardo Palacios Valladares, Pamela Michelle Cornejo Rivas, Melissa S. Nolan

**Affiliations:** 1 Arnold School of Public Health, University of South Carolina, Columbia, SC 29208, USA; kyndallb@email.sc.edu (K.C.D.-B.); hewaltz@email.sc.edu (H.W.); lynnmk@mailbox.sc.edu (M.K.L.); 2 Department of Medicine, University of Arizona College of Medicine, Tucson, AZ 85719, USA; sklotz@email.arizona.edu; 3 Southwestern Biological Institute, Tucson, AZ 85719, USA; ponerine@dakotacom.net; 4 Department of Entomology, Plant Pathology and Weed Science, New Mexico State University, Las Cruces, NM 88003, USA; aromero2@nmsu.edu; 5 Centro de Investigación y Desarrollo en Salud, Universidad de El Salvador, San Salvador, El Salvador; marvin.rodriguez@ues.edu.sv (M.S.R.A.); jose.palacios4@ues.edu.sv (J.R.P.V.); pampdc@hotmail.com (P.M.C.R.)

**Keywords:** *Triatoma rubida*, *Triatoma protracta*, *Triatoma recurva*, mites, ectoparasites, *Trypanosoma cruzi*, Chagas disease, United States

## Abstract

**Simple Summary:**

An estimated 70 million persons in the Western Hemisphere are living at risk for Chagas disease, a parasitic infection transmitted to humans by over 156 different competent triatomine insect vector species. Prior Pan American Health Organization insecticide campaigns throughout Latin America in the 1990s and 2000s demonstrated that domestic insecticide spraying had temporary effects, which resulted in the re-establishment of triatomine species within a few years. Serendipitously, our team found ectoparasitic mites parasitizing triatomines collected from the field in multiple locations in the southwestern United States, where human–triatomine interaction was high but human parasite infection remains low. Upon further investigation of 408 triatomines collected across multiple field sampling sites in Arizona and New Mexico, 13% were found to be parasitized by mites. Mites were found on both *Triatoma rubida* and *Triatoma protracta* species and corporally dispersed on the head, thorax, abdomen and legs of these species. Interestingly, there was no statistical difference in *Trypanosoma cruzi* infection status between parasitized and unparasitized triatomines. Upon further review of the scientific literature, two Latin American-based studies suggest that the presence of mites on triatomines might reduce vector competency via decreased fitness and fecundity. This study provides the first contemporary report of triatomine ectoparasitism, which warrants further investigation as the biologic role of this host-attached mites on *Trypanosoma cruzi* transmission efficacy.

**Abstract:**

Background: Chagas disease is a leading cause of cardiac failure in Latin America. Due to poor safety profiles and efficacy of currently available therapeutics, prevention is a priority for the millions living at risk for acquiring this clinically important vector-borne disease. Triatomine vectors of the Chagas disease parasite, *Trypanosoma cruzi,* are found in the southwestern United States, but risk for autochthonous transmission is thought to be low. The role of ectoparasitic mites is under-explored regarding the ecology of triatomines and Chagas disease transmission. Methods: Triatomine collections were performed using three common entomologic techniques in 2020–2021 from four different locations in southern Arizona and New Mexico. Triatomines were analyzed visually under a 112.5× microscope for the presence of externally attached mites. Following mite removal, triatomines were tested for *T. cruzi* infection by PCR. Results: Approximately 13% of the collected triatomines had mites securely attached to their head, thorax, abdomen, and legs. More than one mite attached was a common finding among ectoparasitized triatomines. Mite presence, however, did not statistically influence triatomine *T. cruzi* status. Conclusions: Our findings add to a growing body of literature demonstrating the sustainability of mite-infested triatomine populations throughout the Western Hemisphere. Future investigations are warranted to better understand the biologic impact of triatomine mites and their potential to serve as a potential biological control tool.

## 1. Introduction

*Trypanosoma cruzi*, the etiologic agent of Chagas disease, is predominately transmitted by 156 members of the Triatominae subfamily in the Western Hemisphere [1,2]. An estimated 70 million individuals are currently at risk of infection globally [3], including in the southern United States [4,5], due to failed insecticide campaigns. Prior insecticide campaigns in Latin America, including the Southern Cone Initiative from the 1990s to 2000s, have provided only temporary solutions due to insecticide resistance of target species and the resulting presence of several key peridomestic competent vectors that have replaced initially eliminated species [6,7]. After infection, acute disease may occur, which is typical in children. Symptoms of acute disease include fever, tachycardia, and encephalomyelitis, and without treatment, 5–10% of those experiencing acute symptoms will die [8]. Additionally, for those infected earlier in life, lifelong infection can occur if undiagnosed and untreated. The chronic phase of Chagas disease can cause nonischemic dilated cardiomyopathy in up to one-third of those infected in the United States and Latin America [5]. Once chronic infection progresses to symptomatic disease, anti-trypanosomal therapy is less effective, highlighting the value of prevention and early identification [7,9]. In addition, significant side effects associated with available therapeutics lead to low patient adherence to the full course of treatment [10,11] and poor treatment efficacy [5,12], making prevention the optimal public health strategy and warranting the development of novel control methods. 

*T. cruzi* is a kinetoplastid protozoan characterized by rapid reproduction in the midgut of the triatomine vector and ultimate migration to the triatomine hindgut, where metacyclic trypomastigotes are released in triatomine fecal material, eventually entering circulation with the ability to infect hosts on which the triatomine vector feeds [4]. In the United States, eleven triatomine vectors are present, with varying prevalence rates of *T. cruzi* infection [4]. Previous research in West Texas has identified certain domestic regions that exhibit low human infection rates despite ahigh prevalence of *T. cruzi* in the triatomine population and high rates of human–triatomine interactions [13]. In an ecologically similar location in Arizona, over 2200 total bites were reported by 105 individuals, none of whom exhibited detectable *T. cruzi* levels, despite the presence of *T. cruzi*-infected triatomines in their domiciles [14]. 

There are eleven triatomine species native to the United States: Paratriatoma hirsuta, *P. lecticularia*, *Triatoma*. *gerstaeckeri*, *T*. *incrassata*, *T*. *indictiva*, *T. neotomae*, *T. protracta*, *T. recurva*, *T. rubida*, *T. rubrofasciata* and *T. sanguisuga*, eight of which serve as competent vectors for *T. cruzi* [4,15,16]. Of the United States’ native species, natural infection of *T. cruzi* has been found in all except for *T. incrassata* and *P. hirsute* [4]. Ecologically, triatomines are nest parasites, targeting small nesting mammals such as woodrats; however, in portions of the southwestern United States, triatomine intrusion into domestic structures and feeding on human inhabitants have been documented [14,15]. Construction materials such as adobe and substandard housing in impoverished U.S. communities contribute to the triatomine infestation of human homes, where vectors are able to survive and reproduce. In the southwestern United States, mammalian hosts include coyotes, raccoons, and other small rodents, whereas primary mammalian hosts in the southeastern United States include opossums, armadillos, racoons, and skunks [4]. A wide range of competent reservoir species have been documented, contributing to the continued sylvatic cycle of transmission across the nation [16]. 

To the best of our knowledge, biological control has not been thoroughly considered for combating the transmission of this disease. Certainly, triatomines are preyed upon by mammals [17]; however, nothing has been noted in the literature about the possibility of a true ecological biological control method. Serendipitously, our team found ectoparasitic mites parasitizing triatomines collected from the field in multiple locations in the southwestern United States where human–triatomine interaction is high, but *T. cruzi* parasite transmission remains low. A literature review revealed a handful of prior research articles describing mites found on triatomines in the United States, mostly before the 1970s [13,18,19,20]. Additionally, two studies from Chile and Mexico present similar mite–triatomine infestation studies on two triatomine species found in their respective countries [21,22]. A doctoral dissertation from 1968 described ectoparasitic mites on five different triatomine species in the United States, but little data can be found in the contemporary literature about the role these mites may play as a biological control mechanism [18]. Mites may serve a purpose in decreasing the triatomine vector fitness and fecundity [22,23,24]. The objectives of our current investigation were to ascertain the presence and corporal distribution of ectoparasite mites on triatomines collected from Arizona and New Mexico, to determine the relationship between mite presence and triatomine *T. cruzi* infection status. 

## 2. Materials and Methods

Collections of triatomines were conducted at multiple sites in Arizona and New Mexico during the summer of 2020. Additionally, one of the original collection sites mailed in additional triatomines to the University of South Carolina laboratory in summer 2021. Triatomines were collected on either private property with permission from the owner or at AZ State Parks with research permission (permit # MULTI-003, dated 8 June 19). An ultraviolet (UV) light was used to attract triatomines at night through sheetlighting in both Tucson and Bisbee (Pima and Cochise Counties, respectively), AZ, and Silver City (Grant County), NM. Sheetlighting was conducted from May to August in Tucson and once in August 2020 in Bisbee and Silver City. Triatomines were collected from the adjacent area and sheet during sheetlighting. In Pima County only, woodrat nests (*Neotoma* spp.) were excavated and searched for triatomines during the early morning hours in August 2020. All collected triatomines were stored in 70% ethanol at room temperature until microscopic examination and diagnostic testing. Additionally, fibrous woodrat nesting material and soil were collected to facilitate alternative collections of potential ectoparasitic mites or triatomine eggs. Finally, triatomines were collected from residents at three sites on non-sheetlighting nights when these triatomines entered homes without a UV light. These triatomines were also stored individually in 70% ethanol until study staff received these. Additional triatomines were submitted to our laboratory in June 2021 from Grant County, NM, one year after the original collections; these were stored at −20 °C until shipped. 

All adult triatomines were identified to species morphologically using a dichotomous key [23]. Sex was also determined and recorded. Nymphal triatomines were not identified to species or nymphal instar morphologically. All triatomines were examined for ectoparasitic mite presence and distribution using a stereomicroscope (Nikon SMZ1500 equipped with a Nikon WD 54 1X objective and Nikon C-W10xA/22 eyepieces, Nikon Inc., Minato City, Tokyo, Japan). Mites were removed using two round, synthetic, size 0 paintbrushes (Michaels Stores, Inc., Irving, TX, USA) and placed in 70% ethanol in separate 1.5 mL microcentrifuge tubes. Mite species identification was attempted, but validation was not successful. 

In total, 403 triatomines were collected; however, only 393 triatomines were analyzed for the presence of *T. cruzi* parasite DNA by PCR, using previously published methods [25]. Ten triatomines were poorly preserved following a delay in shipment (submitted to our laboratory after the initial collections), effectively drying out the triatomines and not adequately preserving the gut contents for analysis. Triatomines were cut at the mesothorax, and tissue was extracted from the hindgut of triatomines to obtain parasite DNA, using QIAGEN DNeasy Blood and Tissue Kits ^®^ (QIAGEN, Hilden, Germany). Go Taq ^®^ Green Master Mix (Promega, Madison, WI, USA) was used for amplification with primers 121 and 122 to amplify highly conserved regions of the triatomine kinetoplast [24]. DNA was denatured at 94 °C for 3 min, followed by 35 cycles at 30 s at 94 °C, 30 s at 55 °C, 30 s at 72 °C, followed by a final extension for 10 min at 72 °C. 

To ascertain whether or not mite presence was associated with *T. cruzi* infection in triatomines, a chi-squared test and a one-way ANOVA were conducted in SAS (SAS v9.4, SAS Institute, Cary, NC, USA).

## 3. Results

**Figure 1:**
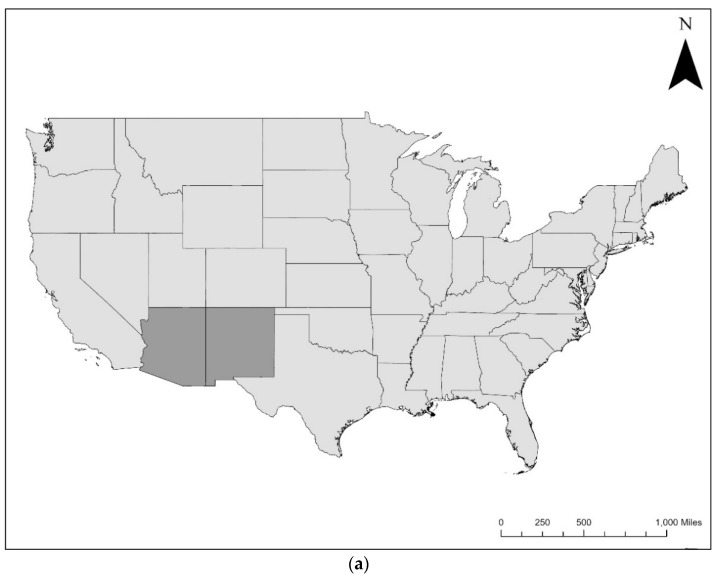

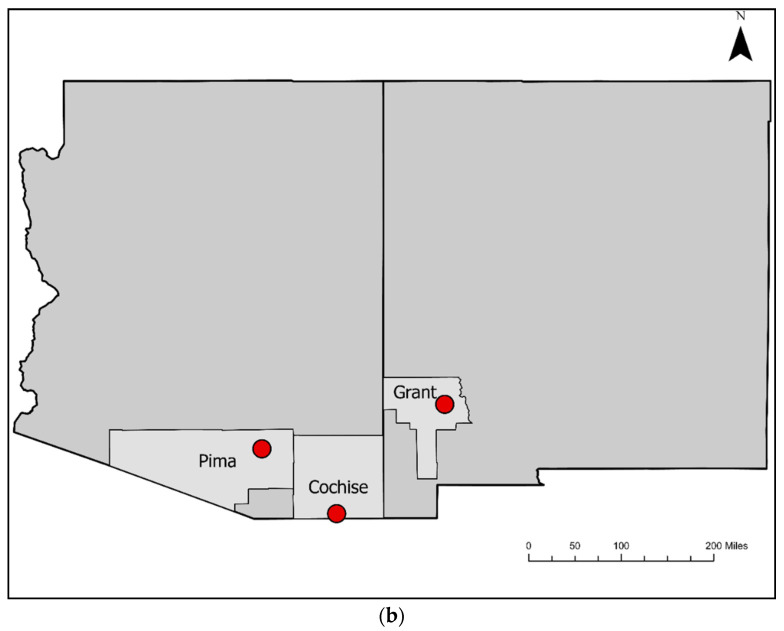
Triatomine collection sites in southern Arizona (Pima and Cochise Counties), and southern New Mexico (Grant County). Both states are located in the southwestern contiguous United States, shown in dark grey (**a**). Mites were found on triatomines from both Pima County, AZ, and Grant County, NM, indicated by red circles (**b**).

From convenience sampling collections in spring–summer 2020 and June 2021, a total of 403 individual specimens were collected at four field collection sites in three counties across southern Arizona and New Mexico (Figure 1). In Pima County, AZ, 380 triatomines were collected (*n* = 367 triatomines were self-collected by the study team through sheetlighting, and *n* = 13 triatomines were collected through woodrat nest excavation by the study team). In Cochise County, AZ, a total of 10 triatomines were collected (all from self-collections from residents; no triatomines were collected by the study team through sheetlighting). In Grant County, NM, 13 triatomines were collected (*n* = 10 were self-collected by residents, and *n* = 3 were collected by the study team through sheetlighting). Ectoparasitic mites were found on triatomines collected in both Pima County, AZ, and Grant County, NM. No mites or triatomine eggs were found in any of the fibrous woodrat nesting material or nearby soil from Pima County from woodrat nest excavation activities. 

Three species and a mix of adult versus immature nymphs were collected (Table 1). *Triatoma rubida* was the most commonly collected species (*n* = 314), followed by *T. protracta* (*n* = 68), *T. recurva* (*n* = 7), and unidentified triatomine nymphs (*n* = 14). The majority of collected triatomines originated from Pima County, AZ (*n* = 380), followed by 13 from Grant County, NM, and 10 from Cochise County, AZ. Overall, 17.6% of triatomines tested were *T. cruzi*-positive. Adult triatomines were more likely to be *T. cruzi*-positive, with only a single immature nymph collected from a woodrat nest having tested *T. cruzi*-positive. Finally, all positive triatomines were collected from Pima County, AZ. 

Overall, 13.2% of collected triatomines were ectoparasitized with mites. Of the parasitized triatomines (*n* = 53), 10.7% were *T. cruzi*-negative, compared with 2.0% parasitized *T.* *cruzi*-negative triatomines. This difference was not significant (*p* = 0.7567). Among triatomines parasitized with mites that were analyzed for *T.* *cruzi* (*n* = 50), the average number of mites did not differ between triatomines positive for *T. cruzi* (F-value = 1.80, *p* = 0.1860), nor did the average number of mites differ between male and female triatomines (F-value = 0.06, *p* = 0.8135). 

**Figure 2:**
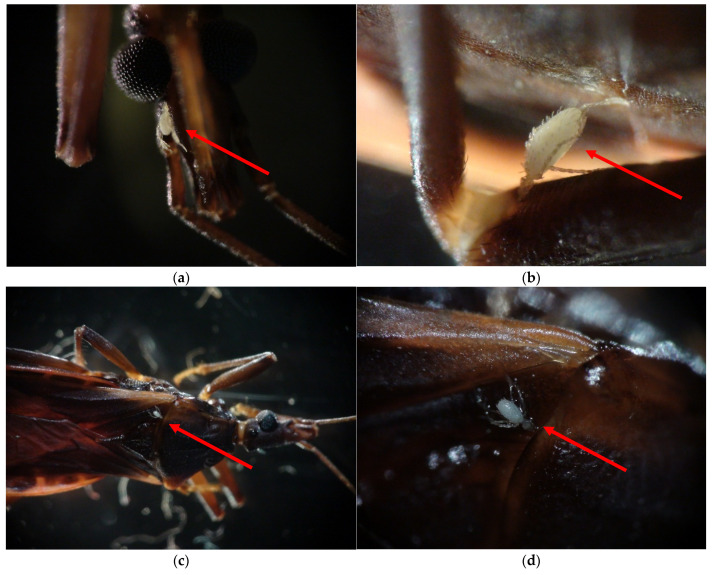
Mite locations on triatomines. Red arrows highlight the presence of mites on *T. rubida* specimens: locations included the (**a**) head (40× magnification); (**b**) legs (112.5× magnification); (**c**) and (**d**) thorax (15× and 45× magnification, respectively).

Mites were found at various attachment sites on their triatomine hosts (Table 2). A more varied distribution of ectoparasitic mite location on triatomine hosts was observed in *T. rubida* samples than in *T. protracta* samples. Of the *T. rubida* triatomines hosting mites, the range was 1–6 mites per triatomine, compared to a range of 1–4 on *T. protracta*. Of the seven *T. protracta* with mites, all exhibited mites solely on the abdomen and the legs. The average number of mites per *T. protracta* was 1.71. A total of 45 *T. rubida* samples were observed to have ectoparasitic mites, and the distribution was much greater, with mites observed on the head, thorax, abdomen, and legs, as shown in Figure 2. It is important to note that 14 mites had become detached from their respective triatomines while in the ethanol-filled vials, and as such, it was impossible to determine their initial points of attachment.

## 4. Discussion

To the best of our knowledge, this is the first contemporary report of mite ectoparasitism of triatomine insects in the United States. The United States is not considered an endemic country for Chagas disease; however, autochthonous cases from local triatomine species do occur [25]. An ongoing scientific debate between local entomologists has resulted in the hypothesis that autochthonous *T. cruzi* transmission does not occur in the United States due to the principle triatomine species’ preference to defecate post-blood meal away from the human host, resulting in a significant reduction in transmission potential [26]. However, this hypothesis is controversial, with some experts having provided counter evidence [27]. The current findings highlight an alternative hypothesis as to why triatomines in this region of high vector diversity and abundance [28] do not effectively yield human infection. Although our findings are descriptive in nature, they add to a growing body of scientific literature on triatomine ectoparasitism, which includes laboratory-based vector competency studies.

Ectoparasitic mites were first observed on the Chagas disease vectors in 1944, and relatively few publications have described their relationship with triatomines since the 1970s [29,30,31]. Most reports describe mites attached and feeding from triatomines, and one report has described mites utilizing triatomines as a vehicle of transportation through phoresy [31]. The most prominent historical study on ectoparasitic mites was from an Indiana entomology doctoral student who published an extensive multi-state triatomine ecology study in which the presence of *Pimeliaphilus* spp. mites was described on *Triatoma rubida*, *Triatoma protracta*, *Triatoma gerstaeckeri*, *Triatoma sanguisuga* and *Paratriatoma hirsuta* [18]. Similar to our findings, that investigation also revealed that adults and older instars were more likely to have mite presence and first instars were completely devoid of mites [18], suggesting that mite presence is acquired following the first molt. Laboratory-based colony biology studies suggest that the ectoparasitism of triatomines is trans-stadial, with female mites becoming permanent fixtures on their host [19]. 

Given the historical nature of prior publications, our current proposal is the first to evaluate the association between *T. cruzi* PCR positivity and ectoparasitic mite presence. However, we did not find a significant association between triatomines infested with ectoparasitic mites and *T. cruzi* infection. Epimastigotes replicate in the hindgut of the triatomine and not in the insect hemolymph; therefore, it is biologically unlikely that mites impact the replication of *T. cruzi* once inside the triatomine host [13,18]. In contrast, there is evidence which suggests that mites may serve as a mechanical means in decreasing the triatomine vector viability [22,23,25], thus lowering the overall *T. cruzi* transmission potential among an ectoparasitized triatomine population. Ectoparasitic mites on triatomines and other insects have been shown to significantly impact fitness, leading to reduced egg production, longer molting times, inviable eggs, and even population decimation in laboratory colonies [22,23,24,32,33]. However, one conflicting report exists in which one sentence states, “ectoparasitic mites have been observed on *Triatoma rubida* but did not appear to interfere with development” [32]. That study, however, was in regard to elucidating the optimal laboratory conditions for *Triatoma* colony establishment, and these mechanical environmental conditions likely influenced the lack of ectoparasitic–host effects. Finally, our *T. cruzi*-mite statistical analysis should be interpreted with caution because the relatively low sample size (*n* = 403) and small number of collection sites (*n* = 4) yielded poor statistical power. Although our sample size was relatively low, the percentage of parasitism in *T. rubida* (14.6%) and *T. protracta* (10.3%) fell within the range or were close to the documented parasitism rate of these species: 2.5–25% for *T. rubida* and 14.3% for *T. protracta* [13,20,34]. 

A primary limitation of the current finding was our team’s inability to speciate the ectoparasitic mites. Previously published research documents two genera that parasitize triatomines: *Leptus* (Trombidiformes: Erythraeidae) in South America and *Pimeliaphilus* (Trombidiformes: Pterygosomatidae) in North and South America [22,30]. Nine mite species have been reported, seven of which fall under the genus *Pimeliaphilus* [22,30]. In total, 12 species of triatomines have been documented as parasitized by ectoparasites in 6 countries [30]. In the United States, triatomines infested with mites have been reported in Arizona (Pima County), California (Kern, Riverside, and San Bernadino Counties), Texas (Gillespie and Brewster Counties) and in Indiana (Dubois County), with the majority of collection reports coming from Prima County, AZ [13,20,22,34]. The current manuscript is the first report of ectoparasitic mites found on triatomines from New Mexico (Grant County). Historically, *T. rubida* has been found parasitized by *P. plumifer* only, whereas *T. protracta* has been documented naturally infested with both *P. plumifer* and *P. calimesae* in the southwestern United States [20]. Of the counties in our study, Pima County, AZ, was the only one with a history of documented parasitized triatomines, specifically *T. rubida, T. protracta,* and *T. recurva* [18,20]. Given this contextual information, we hypothesize that the mites found on our collected triatomines were from the *Pimeliaphilus* genus. Identifying these ectoparasitic mites would help to elucidate the role of mites on triatomine fitness and ultimately how parasitized triatomines’ vector capability is impacted.

Although we were unable to speciate the mites, this manuscript is the first publication to provide photographs of triatomine ectoparasitizing mites, making Figure 2a–d important for informing the wider scientific community. Furthermore, the majority of prior publications did not report the corporal distribution of mites on triatomines, as discussed in Table 1. Our evidence suggests that mites are most commonly found on the legs, but it is not uncommon to find mites attached at either the abdomen or the thorax. In *T. rubida* samples, we identified an average of 1.56 mites per triatomine (*n* = 45). In *T. protracta* samples, a slightly higher average of 1.71 mites was found per triatomine (*n* = 7). However, the smaller sample size of *T. protracta* may contribute to decreased precision of the results. 

These findings are a serendipitous convenience sampling; therefore, more data on mite prevalence found on triatomines in the southwestern United States is needed to validate the regional establishment of these entomologic populations. Triatomines have long been described as allergenic pests of the southwestern United States, and have previously been collected from all three selected counties described in this study [33,35]. The contemporary finding of mites ectoparasitizing triatomines from well-established endemic countries begs the question of their relevance and temporal establishment. Furthermore, future studies are warranted to both determine the precise role of mites in triatomine fitness and to elucidate their presence in highly endemic Latin American countries. Few studies have explored mite presence on triatomines in Latin America: infested triatomines have been reported in Costa Rica, Mexico, Argentina, Chile, and Peru [22,24,33,36]. One report noted a *Pimeliaphylus* parasitism rate of over 53% on collected triatomines in Costa Rica, demonstrating that infestation rates can reach relatively high rates in non-laboratory conditions [33]. Co-authors P.M. Cornejo Rivas and M. S. Rodriguez Aquino also report collected triatomines infested with ectoparasitic mites from El Salvador (unpublished data). Another key factor in investigating mite prevalence and impacts on vector capability could be environmental factors such as domestic vs. sylvatic environments, elevation, and climate. Investigating mite presence in human habitation-collected triatomines in an endemic region could yield answers to the overall question of how these ectoparasitic mites affect triatomines in relation to public health. Future exploratory studies are needed to provide contemporary data to better identify the ecological role of these mites and their potential application in public health directives.

## 5. Conclusions

Chagas disease is a leading cause of nonischemic cardiomyopathy and heart failure in Latin America, and hundreds of thousands of infected individuals are thought to reside within the United States. Autochthonous transmission is possible, albeit rare, in the United States from native triatomines, some of which have been found with ectoparasitic mites. Current therapeutics are ineffective once disease progression has begun, and the only currently available preventative insecticide method is temporary; therefore, new mechanisms for vector control are warranted. This manuscript provides the first contemporary evidence of triatomine ectoparasites in a region of high vector–human contact, although low rates of transmission. Mites were present on a two triatomine species captured from multiple sites in southern Arizona and New Mexico, which demonstrated varied corporal dispersion and were found to polyparasitize their host. Further epidemiologic studies are warranted to better understand the biologic role of mites on triatomine fitness and the geographic expansion of this entomologic phenomenon. 

## Figures and Tables

**Table 1:** Mite parasitization and *T. cruzi* infection rates of *Triatoma* sp. collected in Arizona and New Mexico.

Species	Number	Parasitized by Mites (%)	*T. cruzi*-Positive (%)
*Triatoma rubida*	314	46 (14.6%)	54 (17.2%)
*Triatoma protracta*	68	7 (10.3%)	14 (24.1%) ^1^
*Triatoma recurva*	7	0 (0.0%)	0 (0%)
Immature nymph	14	0 (0.0%)	1 (7.1%)

^1^: This percentage is 14 of 58 *T. cruzi* analyzed *T. protracta.* Ten *T. protracta* were not at a sufficient quality for PCR testing.

**Table 2:** Mite location and count on *Triatoma rubida* and *Triatoma protracta* specimens (*n* = 53).

Species (No.)	Avg. No. Mites per Triatomine	Head	Thorax	Abdomen	Legs	Unknown ^1^
*Triatoma rubida* (46)	1.56	4	14	18	25	10
*Triatoma protracta* (7)	1.71	0	0	3	4	4

^1^: Some ectoparasitic mites were found inside the vial, already unattached from the triatomine. We were unable to determine the attachment site for these mites.

## Data Availability

Data are available upon reasonable request.
